# Development and evaluation of enzyme-linked immunosorbent assay of nucleic acid sequence-based amplification for diagnosis of invasive aspergillosis

**DOI:** 10.1186/s13568-016-0266-0

**Published:** 2016-10-06

**Authors:** Li Du, Yun Xia, Yunyan He, Qingquan Pu, Ruoyi Hua, Wenyao Wu

**Affiliations:** 1 Department of Laboratory Medicine, the First Affiliated Hospital of Chongqing Medical University, Chongqing, 400016 People’s Republic of China; 2 Department of Laboratory Medicine, Chongqing People’s Hospital, Chongqing, 400016 People’s Republic of China

**Keywords:** NASBA-ELISA, RT-PCR, GM-EIA, Invasive aspergillosis

## Abstract

Invasive aspergillosis (IA) is a life-threatening infection in immunocompromised patients, rapid and sensitive detection of *Aspergillus* from clinical samples has been a major challenge in the early diagnosis of IA. An enzyme-linked immunosorbent assay of nucleic acid sequence-based amplification (NASBA-ELISA) was developed to fulfil the need for the efficient diagnosis of these infections. The primers targeting 18S rRNA were selected for the amplification of *Aspergillus* RNA by the isothermal digoxigenin (DIG)-labeling NASBA process. The DIG-labeled RNA amplicons were hybridized with a specific biotinylated DNA probe immobilized on streptavidin-coated microtiter plate. The hybrids were colorimetrically detected by the addition of an anti-DIG antibodies linked to ALP and substrate (disodium 4-nitrophenyl phosphate). The detection limit of the *Aspergillus* NABSA-ELISA system was 1 CFU and the RNA in non-target bacteria or fungus was not amplified. The performance of this NASBA-ELISA compared to RT-PCR and galactomannan (GM) was evaluated by testing blood samples from 86 patients at high risk for IA. The sensitivity of NASBA-ELISA, RT-PCR and GM-ELISA was 80.56 % (95 % CI 63.98–91.81), 72.22 % (95 % CI 54.81–85.80), 58.33 % (95 % CI 40.76–74.49), respectively, and the specificity was 80.00 % (95 % CI 66.28–89.97), 84.00 % (95 % CI 70.89–92.83), 82.00 % (95 % CI 68.56–91.42). The efficiency of the three methods in various combinations was also evaluated. Combination of NASBA-ELISA and GM-ELISA testing achieved perfect specificity (100 %; 95 % CI 92.89–100) and perfect positive predictive value (100 %; 95 % CI 83.16–100). The best sensitivity (97.22 %; 95 % CI 85.47–99.93) and the highest Youden index (0.652) were obtained by testing with both NASBA and RT-PCR in parallel. In conclusion, the NASBA-ELISA assay consists of an alternative process for large-scale samples detection with semi-quantitative results and provides good clinical performance without resorting to expensive equipment. This assay makes it possible for the NASBA based RNA diagnosis to become a routine work in laboratories in less developed countries with fewer resources.

**Electronic supplementary material:**

The online version of this article (doi:10.1186/s13568-016-0266-0) contains supplementary material, which is available to authorized users.

## Introduction

Invasive aspergillosis (IA), an opportunistic fungal infection, is increasingly recognized as a major cause of morbidity and mortality in immunocompromised patients, including those receiving aggressive chemotherapy or immunosuppressive drugs (Chandrasekar 2010; Barton 2013; Leventakos et al. 2010). Because of its high mortality, early diagnosis is the key to successful treatment. However, microbiological examination such as *Aspergillus* culture is time-consuming and has lower positive rate (Torelli et al. 2011), it is also difficult in distinguishing between infection and colonization. Diagnosis by biopsy or histopathology is difficult due to the severity of illness in these patients. Galactomannan by enzyme immunosorbent assay (GM-EIA) is one of the standard measures (Yoo et al. 2007; Boutboul et al. 2002) and is included as an important component of microbiological factors in the diagnostic criteria devised by the Mycology Study Group of the European Organization for Research and Treatment of Cancer (EORTC/MSG) (De Pauw et al. 2008). Performance of GM-EIA can vary considerably and has been shown to be affected by various factors, leading to both false positive and false-negative results (Mikulska et al. 2012). Galactomannan is not unique to *Aspergillus* spp., there are cross-reacting antigens in other fungus and some antibiotic agents. Antibody detection methods often cannot distinguish between current and past infection and it performs poorly in patients with haematological malignancy or having undergone bone marrow or stem cell transplantation.

More recently, nucleic acid amplification tests (NAATs) in vitro can provide increased sensitivity and specificity of detection (van der Vliet et al. 1993; Ieven and Goossens 1997). Nucleic acid sequence-based amplification (NASBA) is a molecular method for amplification of target RNA segments in which the necessity of thermo cycling is excluded due to lack of any thermal variation in the reaction environment. The amplification efficiency of NASBA is more robust than that of PCR (Mercier-Delarue et al. 2014), yielding more than 10^12^ amplicons in as little as 30 min (Zhao et al. 2009). The advantages of NASBA have promoted interest in evaluating its application to detection of *Aspergillus* RNA in clinical samples. The main drawback of NASBA is that special care is needed in sample preparation and detection, due to the profound susceptibility of RNA to degradation. Thus, further studies are warranted to improve the ability to quantify pathogen load and develop automated, standardized protocols for sample processing and data analysis. Conventional NASBA requires several steps after RNA amplification, including electrophoresis or blotting and hybridization, which are inconvenient for detecting a large number of samples in clinical routine. The NASBA-enzyme-linked immunosorbent assay (NASBA-ELISA) consisting of an alternative process for large-scale screening allows for application in daily life. This technique combines an immunological method to quantify the NASBA product directly after immobilization of biotinylated DNA on a microplate. The NASBA-ELISA can be performed without special equipment and reagents for detection of amplified product compared to NASBA-electrophoresis. Therefore, NASBA-ELISA allows the using of RNA based diagnosis for routine purposes in poor developed laboratories.

The aim of this work was developing an NASBA- ELISA system as an improvement over traditional assays to detect *Aspergillus* 18s RNA in human blood samples for diagnosis of invasive aspergillosis. RT-PCR and GM test were performed simultaneously to evaluate the performance of the three methods.

## Materials and methods

### Fungal and bacterial strains

*A. fumigatus* strain CMCC A1a, kindly provided by Dr. WeiPing Lu (Daping Hospital, Third Military Medical University of People, Liberation Army, ChongQing, China), was grown on Sabouraud’s dextrose agar at 37 °C for 2–3 days. The number of *Aspergillus* spores diluted in saline suspension was quantified using a Neubauer chamber, and a solution containing approximately 10^5^ spores was used for total RNA extraction. Strains of *E.coli* ATCC25922, *Candida albicans* ATCC64548, *Staphylococcus aureus* ATCC29213, *Pseudomonas aeruginosa* ATCC27853 and *Cryptococcus neoformans* FY226 (isolated from clinical samples) were cultured on blood agar at 37 °C for 1–2 days. The total RNA from these strains was extracted using Trizol reagent (Invitrogen, California, America) strictly following the manufacturer^`^s instruction.

### Patient populations and blood samples

The definitions of invasive fungal infection according to the EORTC/MSG (De Pauw et al. 2008) updated criterion are classified into “proven”, “probable” and “possible”. To define as a proven invasive fungal infection it is required that a fungal hyphae or spores be detected by histological analysis or in the culture of a specimen obtained by a sterile procedure from a normally sterile and clinically or radiologically abnormal site consistent with an infectious disease process of tissue taken from the infected site of the disease. Probable invasive fungal infection requires the presence of a host factor, a clinical criterion, and symptoms consistent with the disease entity, and mycological evidence including: direct microscopic analysis, culture and serologic test such as GM.

Based on this criterion, a total of 36 patients at high risk for IA (including 9 proven cases, 27 probable cases) were served as test group and 50 patients not achieving an EORTC/MSG diagnosis criterion were served as control group. Cases were selected on the solid basis of sample availability and diagnosis, whereas controls were randomly selected from a period of testing similar to that of the IA cases. All patients were enrolled from January 2014 to December 2015 in the First Affiliated Hospital of Chongqing Medical University, a 3200-bed teaching hospital in Chongqing, southwestern China. Information of patients was retrieved from the hospital information system and the laboratory information system (Additional file 1). Plasma samples were collected from the samples that were sent to our department for (1,3)-β-D-glucan and galactomannan detection before any antifungal therapy began. Plasma was separated from each fresh blood sample by centrifugation at 2500×*g*, transformed to Rnase free tubes and stored at −80 °C for further use.

### *Aspergillus* GM detection

Platelia kits (Bio-Rad Laboratories, Hercules, CA) were used to measure the galactomannan level in blood samples. Assay performance and interpretation of positivity were performed by an investigator blinded to case status and as directed by the manufacturer. Optical densities were determined using a threshold index (optical density at 450/620 nm [OD_450/620_] of sample/OD_450/620_ of threshold control) with the Sunrise Microplate Reader (Tecan, Männedorf, Switzerland). Samples were run with positive, negative, and threshold controls supplied by the manufacturer. Optical density index was calculated as the optical density of the sample divided by the mean optical density of 2 threshold controls. On these grounds, any value above 0.5 was considered positive.

### RNA extraction

In terms of determining analytical sensitivity the NASBA-ELISA assays, briefly, fungal spores suspensions of a certain concentration (10^6^/ml, 10^5^/ml, 10^4^/ml, 10^3^/ml) were prepared by microscope count method using Neubauer’s hemacytomete. 100 μL portions of fungal suspensions which had been grinded in mortar (free of Rnase) with liquid nitrogen were used for RNA extraction by total RNA rapid extraction kit. In terms of clinical application on the NASBA-ELISA, 200 μL of plasma samples (restored to room temperature from −80 °C) were used for RNA extraction by the same kit as above. The above samples mixed 300 μL of RLS lysis buffer followed by incubation for 10 min at room temperature. The tubes were vortexed vigorously for 15 s and then incubated for 3 min after addition of 150 μL chloroform. The aqueous phase was transferred to a spin column AC and mixed with 500 μL of 70 % ethanol, followed by  centrifugation at 12,000×*g* for 10 min at 4 °C. The RNA was washed sequentially with buffer RE and buffer RW. Finally, 60 μL eluate was obtained and stored at −80 °C until further use. (low concentration of RNA was obtained through tenfold serial dilutions of RNA extracted with high concentrations of spores).

### Primers and probe

A primer pair encoding a highly conserved 18S rRNA region specific for the *Aspergillus* genus was chosen as previously reported to amplify a 243 nucleotide fragment of the target RNA that includes the species-specific region for oligonucleotide probe to identify the *Aspergillus* spp. (Loeffler et al. 2001). The reverse primer (Primers1.2) bears the bacteriophage T7 RNA polymerase promoter binding region and preferred transcriptional initiation sequence at its 5′ end. A biotinylated oligonucleotide probe (hybridization probe) corresponding to an internal region defined by the primers was synthesized for the detection of amplified target RNA (Table 1).

**Table 1:** Nucleotide sequences of oligonucleotide primers and probe used in this study

Primers/probe	Sequence (5′-3′)	Length (bp)
Primers 2.1	5′-GCCGCGGTAATTCCAGCTCCAATA-3′	24
Primers 1.2 + T7^a^	5′-AATTCTAATACGACTCACTATAGGGGAGCAAAGGCCTGCTTTGAACA-3′	47
Hybridization probe	Bioth-GGTCCGCCTCACCGCGAGTACTG-3′	20
NASBA product		243

^a^Underlining indicates the T7 promoter region

### Reverse transcriptase PCR (RT-PCR) process

Reverse transcription was performed in an ABI Veriti™ 96-Well Thermal Cycler (Applied Biosystems; Life Technologies, Carlsbad, CA, USA) with a total reaction volume of 20 μL per well containing 4 μL 5× PrimeScript buffer, 1 μL PrimeScript Enzyme Mix × 1, 1 μL Oligo DT primer (50 μm), 1 μL random 6 mers (100 μm), 5 μL template RNA, 8 μL Rnase water. Thermal cycling conditions were 37 °C for 15 min, followed by 85 °C for 5 s and 4 °C for 5 s and the cDNA product was as template of PCR described below.

PCR reagents in a final volume of 25 μL included: 2 μL template cDNA, 12.5 μL Taq DNA polymerase (5 U/μL), 1 μL of each primer (10 pmol/μL, the same as NASBA primers), 3.5 μL dH_2_O. The PCR program was carried out at 95 °C for 10 min, followed by 35 cycles of 30 s at 95 °C and 30 s at 72 °C. The final extension was at 72 °C for 5 min. PCR products were electrophoresed in 1 % agarose followed by staining with Goldview then visualized under ultraviolet light, and the results were recorded by photography.

### Digoxigenin (DIG)-labeling NASBA process

For DIG-labeling NASBA reaction, *Aspergillus* RNA sample (5 μL) was suspended in 10 μL of amplification reagent solution containing 2 mmol/L each NTP, 0.2 mmol/L Digoxigenin-11-UTP, 1.6 mmol/L each dNTP, 40 mmol/L Tris–HCl (pH 8.5), 70 mmol/L KCl, 5 mmol/L dithiothreitol, 12 mmol/L MgCl_2_, 0.375 mol/L sorbitol, 12 U ribonuclease inhibitor (Promega, Fitchburg, WI, USA), 0.4 μmol/L each primer, followed by an incubation at 65 °C for 5 min and at 41 °C for another 5 min. Then, 5 μL enzyme mixture (40U T7 RNA polymerase (Promega), 2.0 μg bovine serum albumin, 8U avian myelo-blastosis virus reverse transcriptase (Takara Bio), and 0.1 U RNase H) was added. The reaction mixture was incubated for 90 min at 41 °C for isothermal amplification. Negative controls consisted of all of the same reagents but substituting for the target with an equal volume of buffer. The amplification products were analyzed by 1 % agarose gel electrophoresis (Wang et al. 2014; Mollasalehi and Yazdanparast 2013), and by NASBA-ELISA DIG detection system described below.

### Microtiter plate hybridization and detection of NASBA product

Biotinylated probe (Hybridization probe) (0.1 mM, 100 μL) diluted in PBST buffer (phosphate-buffered saline containing 0.05 % (v/v) TWEEN-20, PH 7.2, prepared with DEPC water) was incubated in the streptavidin-coated microtiter plate (restored to room temperature from 4 °C, Haili, JiangSu, China) at room temperature for 1 h with gentle agitation. After aspiration, the plates were washed with 3 × 300 μL PBST buffer to prepare for assay application (Gill et al. 2006). For hybridization, 10 μL DIG-labeled NASBA product was heated 2 min at 95 °C to inactive Rnase H activity and mixed with 90 μL hybridization solution (50 mM sodium-phosphate buffer, pH 7.0, prepared with DEPC water). The mixture (100 μL) was added to the oligoprobe-coated streptavidin microtiter plate and incubated for 1 h at 37 °C with gentle shaking. The plates were washed with 3 × 300 μL PBST buffer. Anti-digoxigenin antibody ALP conjugate (Roche, Basel, Switzerland) diluted 500-fold in PBST buffer was added to each well in a final volume of 100 μL and the microtiter plates were incubated for 30 min at 37 °C. After five washes with PBST solution, 100 μL of developer PNPP (restored to room temperature from −20 °C, Yeasen, Shanghai, China) was added to each well and incubated in the dark at 37 °C for 15 min. Finally, 50 μL of stop solution was added and the developed color was measured at 405 nm in a microtiter plate reader (Ravan and Yazdanparast 2012; Gomes et al. 2010; Di Pinto et al. 2012). All experiments were performed in triplicate with negative controls, and data represent mean values. The cut-off value was calculated as the mean absorbance value of the negative controls plus three standard deviations, as obtained with a single NASBA-ELISA test. As the mean absorbance value and standard deviation were 0.059 ± 0.012, a sample was considered positive when the absorbance of the three measurements was greater than 0.095 (the cut-off value).

### Statistical analysis

Performance parameters for each approach were analyzed by the MedCalc® 15.2.2 and SPSS22.0. Positive results from patients with proven or probable IA according to the EORTC/MSG criteria were considered to be true positives and used to calculate the sensitivity of the three assays. For calculation of specificity, negative results from patients without EORTC/MSG evidence of IA were considered to be true negatives. Receiver operating characteristic curve (ROC) was used to assess the diagnostic value of NASBA-ELISA system. A higher value of area under the curve (AUC) represents a greater diagnostic value of the assay. Comparison between two methods was performed by paired diagnosis test design using Fisher’s exact test to generate two-sided *P* values with a *P* value of ≤0.05 being considered significant. Absorbance readings and arithmetical means of the number of *Aspergillus* spores (transformed into log scale) were analyzed by Pearson’s parametric correlation coefficient or Spearman’s nonparametric correlation coefficient in the NASBA-ELISA test. The Youden index was calculated to evaluate the synthetic ability of each assay. A kappa statistic was determined and interpreted as described previously. Values greater than 0.8 means excellent agreement between methods, values of 0.61–0.8 means substantial agreement, values of 0.41–0.6 means moderate agreement, and values below 0.4 means poor agreement (White et al. 2013).

## Results

### Analytical sensitivity of the NASBA process and analytical sensitivity of the NASBA-ELISA with DIG detection system

**Fig. 1:**
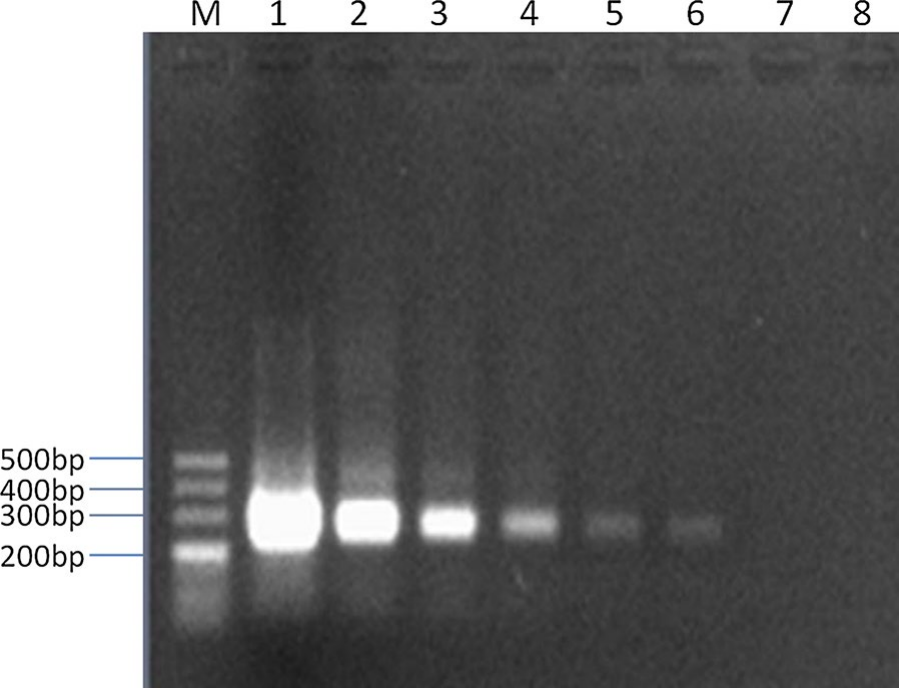
Analytical sensitivity of NASBA followed by 1 % agarose gel electrophoresis with Goldview for tenfold serial dilutions of genomic RNA extracted from a saline solution containing 10^5^ CFU *Aspergillus* spores. *Lane 1* 10^5^ CFU; *lane 2* 10^4^ CFU; *lane 3* 10^3^ CFU; *lane 4* 10^2^ CFU; *lane 5* 10 CFU; *lane 6* 1 CFU; *lane 7* 0.1 CFU; *lane 8*, negative control. The detection limit was 1 CFU

Analytical sensitivity of NASBA by using 1 % agarose gel electrophoresis was obtained by 10-fold serial dilutions of genomic RNA (Fig. 1). The limit of detection was 1 CFU.

**Fig. 2:**
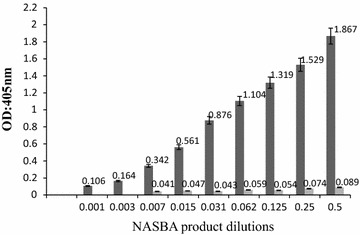
Detection of DIG-labeled NASBA products by the microplate hybridization system. NASBA was performed in the presence of 10^5^ CFU *Aspergillus* spores and then NASBA product was twofold serially diluted before DIG-detection ELISA system (*filled square*). Negative control consists of NASBA reaction performed in the absence of *Aspergillus* (*open square*). Results are averages of triplication analysis

**Fig. 3:**
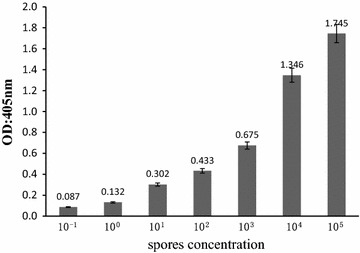
Sensitivity of the NASBA-ELISA for molecular detection of *Aspergillus* 18S rRNA. Tenfold serial dilutions of *Aspergillus* (10^5^ CFU) were prepared and used for 18S rRNA extraction and DIG-labeling NASBA amplification.Then 5 μL of the NASBA product was used in DIG-detection NASBA-ELISA system. Each dilution was analyzed in duplicate

**Fig. 4:**
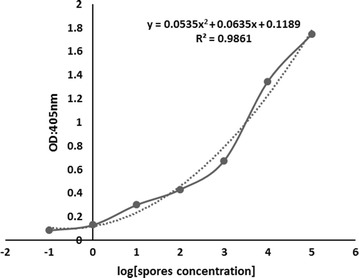
Correlation between log-transformed individual measurements of *Aspergillus* spores and absorbance readings. The determination coefficient between NASBA-ELISA OD and log[spores concentration] was 0.986

The efficiency of the NASBA-ELISA was proved by analyzing two fold serial dilutions of digoxigenin-labeled NASBA amplicons of *Aspergillus*. The OD increased with the increasing concentration of amplification products within a certain range (Fig. 2). Tenfold serial diluted concentrations of nucleic acid sample RNA extracted from a saline solution containing 10^5^ CFU *Aspergillus* spores were employed to determine the sensitivity of the developed method. As the OD_405_ values for 1–10^5^ CFU were above the cut-off threshold value of 0.095 (Fig. 3), the lowest detection was 1 CFU, as the same as that obtained by 1 % agarose gel electrophoresis. The polynomial fitting between the optical density readings of the NASBA-ELISA and the log number of the template spores was significant, with the determination coefficient of 0.986 (Fig. 4).

### Analytical specificity of the NASBA-ELISA with DIG detection system

**Fig. 5:**
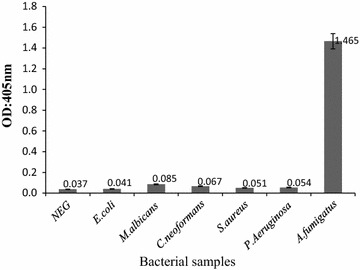
Analysis of specificity of the NASBA-ELISA DIG-detection system. NASBA-ELISA was performed with non-target nucleic acids. NASBA-ELISA was performed on 18S rRNA of *A.fumigatus* as a positive control. DIG-detection ELISA was performed on H_2_O as a negative control. The results are means of triplication analysis

Experimental specificity was assessed by amplifying non-target RNA extracted from *E. coli, C. albicans*, *C. neoformans, S. aureus* and *P. Aeruginosa*. The amplification products were analyzed by ELISA. No positive results were demonstrated for these strains, indicating that the assay is highly specific for *Aspergillus* (Fig. 5).

### Tests results of patients

**Fig. 6:**
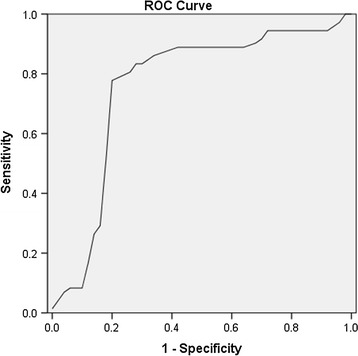
ROC curve of the NASBA-ELISA assay. The ROC analysis is based on optical density values. Area under the curve = 0.760 (95 % CI 0.650–0.870). Diagonal segments are produced by ties

The results of NASBA-ELISA, RT-PCR and GM-ELISA assays were listed in Table 2. The NASBA-ELISA OD values were assessed by ROC analysis (Fig. 6). Area under the curve = 0.760(95 % CI 0.650–0.870). The diagnostic parameters were calculated for each method (Table 3). Comparison of the three assays revealed that NASBA-ELISA had the highest sensitivity, at 80.56 % (95 % CI 63.98–91.81), and RT-PCR had the highest specificity, at 84.00 % (95 % CI 70.89–92.83). Hypothesis testing revealed that sensitivity of NASBA-ELISA was significantly greater than that of GM-ELISA (*P* = 0.023) and no other significant differences were observed among the three tests. In addition, the NASBA-ELISA assay had the best performance in negative predictive value, and Youden index, while RT-PCR was the best performing assay for positive likelihood ratio and positive predictive value. Optimal performance was achieved by comparing the performance of various combinations of the three tests (Table 4, the last page). Perfect specificity (100 %; 95 % CI 92.89–100) and perfect PPV (100 %; 95 % CI 83.16–100) were attainable by combining NASBA-ELISA with GM-EIA in serial testing. Combining NASBA-ELISA with RT-PCR appeared to be better than other combinations for sensitivity, NPV and Youden index.

**Table 2:** Assay concordance on a patient basis

Test combination	No. of population positive by test or test combination	
	Proven/probable (n = 36)	No IA (n = 50)
NASBA-ELISA, *RT-PCR*, GM positive	18	0
NASBA-ELISA, *RT-PCR* positive	2	3
NASBA-ELISA, GM positive	2	0
*RT-PCR*, GM positive	1	1
NASBA-ELISA positive	7	7
*RT-PCR* positive	5	5
GM positive	0	8
NASBA-ELISA, *RT-PCR*, GM negative	1	26

**Table 3:** Performance parameters for GM ELISA, *Aspergillus* RT-PCR, and *Aspergillus* NASBA-ELISA when testing cases of proven/probable IA

Parameter	NASBA-ELISA	RT-PCR	GM-ELISA
Sensitivity [% (95 % CI)]	*80.56 (63.98*–*91.81)*	72.22 (54.81–85.80)	58.33 (40.76–74.49)*
Specificity [% (95 % CI)]	80.00 (66.28–89.97)	*84.00 (70.89*–*92.83)*	82.00 (68.56–91.42)
Positive likelihood ratio	4.03 (2.26–7.17)	*4.51 (2.32*–*8.79)*	3.24 (1.69–6.23)
Negative likelihood ratio	0.24 (0.12–0.48)	0.33 (0.19–0.57)	*0.51 (0.34*–*0.76)*
PPV [% (95 % CI)]	74.36 (57.87–86.96)	*76.47 (58.83*–*89.25)*	70.00 (50.60–85.27)
NPV [% (95 % CI)]	*85.11 (71.69*–*93.80)*	80.77 (67.47–90.37)	73.21 (59.70–84.17)
Youden index	*0.61*	0.56	0.40
κ statistic	*0.60*	0.57	0.41

Combined analysis for both proven IA (n = 9) and probable IA (n = 27)

Best performance values are highlighted in italics

* P < 0.05 versus NASBA-ELISA

**Table 4:** Combined assay performance for GM ELISA, *Aspergillus* RT-PCR, and *Aspergillus* NASBA-ELISA when testing cases of proven/probable IA

parameter	NASBA-ELISA or^a^ RT-PCR	NASBA-ELISA and^b^ RT-PCR	NASBA-ELISA or GM-ELISA	NASBA-ELISA and GM-ELISA	RT-PCR or GM-ELISA	RT-PCR and GM-ELISA
Sensitivity	*97.22 (85.47*–*99.93)*	55.56 (38.10–72.06)	83.33 (67.19–93.63)	55.56 (38.10–72.06)	77.78 (60.85–89.88)	52.78 (35.49–69.59)
Specificity	68.00 (53.30–80.48)	94.00 (83.45–98.75)	62.00 (47.17–75.35)	*100 (92.89*–*100)*	66.00 (51.23–78.79)	98.00 (89.35–99.95)
Positive likelihood ratio	3.04 (2.02–4.57)	9.26 (2.98–28.82)	2.19 (1.50–3.22)	Infinity	2.29 (1.50–3.49)	*26.39 (3.70*–*188.24)*
Negative likelihood ratio	0.04 (0.01–0.28)	0.47 (0.33–0.69)	0.27 (0.13–0.58)	0.44 (0.31–0.64)	0.34 (0.18–0.64)	*0.48 (0.34*–*0.68)*
PPV [% (95 % CI)]	68.63 (54.11–80.89)	86.96 (66.41–97.22)	61.22 (46.24–74.80)	*100 (83.16*–*100)*	62.22 (46.54–76.23)	95.00 (75.13–99.87)
NPV [% (95 % CI)]	*97.14 (85.08*–*99.93)*	74.60 (62.06–84.73)	83.78 (67.99–93.81)	75.76 (63.64–85.46)	80.49 (65.13–91.18)	74.24 (61.99–84.22)
Youden index	*0.652*	0.496	0.453	0.556	0.438	0.508

Combined analysis for both proven IA (n = 9) and probable IA (n = 27)

Best performance values are highlighted in italics

^a^ “or” indicates that at least one of the assays is required to be positive

^b^ “and” indicates that both assays are required to be positive before a patient is considered positive

## Discussion

Invasive aspergillosis (IA) is recognized as an opportunistic infection among many categories of immunocompromised patients (Nabili et al. 2013) and continues to be problematic to diagnose due to the lack of rapid, specific, and sensitive diagnostic methods. The use of NASBA has been reported rarely to detect *Aspergillus* species (Dimopoulos et al. 2012). We developed an NASBA-ELISA assay for analyzing *Aspergillus* RNA in plasma sample to rapidly diagnose IA. ELISA techniques provide an alternative to gel electrophoresis for the detection of NASBA products.

Evaluation of the analytical sensitivity of the *Aspergillus* NASBA-ELISA system showed the same sensitive as NASBA-electrophoresis with the lowest detection concentration of 1 CFU. The high sensitivity of the method is due to two factors: (1) The target of NASBA-ELISA assay, 18rRNA, is present at a level of approximately 100–300 copies in per fungal cell (Park et al. 2011); (2) The Biotin-Avidin-System (BAS) used for immobilization of amplification products on microtiter plates increases the sensitivity of procedure by taking advantage of its multi stage amplification effect.

The NASBA-ELISA system was developed by using primers designed by Loeffler J et al. (2001), who proved specificity of the primers and the use of bioth-probe also assured fine specificity for *Aspergillus* genus. The specificity was also proved by the non-specific amplification signal when non-target RNA was used as templates in our experiment. Further more, a potential risk of contamination with such non-target microbes nucleic acids could be ignored because only the target nucleic acids with T7 promoter can be amplified. Thus, the hybridization signals acquired were comparable in terms of intensity to that of *Aspergillus* alone. As the results indicated, the relation between optical density of ELISA and amplification products concentration was given through polynomial fitting. Once the ELISA reaction has reached saturation, the optical density maybe not be improved obviously by increasing concentration of products. Therefore, NASBA-ELISA assay can be taken as a semi-quantitative technique of amplification products analysis.

GM-ELISA is a broadly used routine diagnostic assay and has been enrolled as a formal component of diagnostic criteria in EORTC/MSG. Reverse transcriptase PCR (RT-PCR), involving treatment with reverse transcriptase enzyme (an RNA-dependent DNA polymerase) prior to a normal PCR, uses the same template RNA as NASBA. Thus, the diagnostic performance of the NASBA-ELISA was compared to RT-PCR and galactomannan (GM) by testing plasma samples simultaneously with the three methods.

It is known that GM testing frequently appeared false-positive results and low sensitivity for patients receiving preventive medication. A previous study has reported that GM is not released into the circulation during infection until the fungus invaded the endothelial compartment (Hope et al. 2007). This suggested that circulating GM could not be detected until angioinvasion by the fungus occurs. Antibodies to GM could also develop which have been suggested as a cause of false negative serum GM results in patients with IA (Herbrecht et al. 2002). It has also been reported that some false positive GM results derived from the existence of GM in some other fungies and in preparations of some antibiotic agents (Vergidis et al. 2012; Tortorano et al. 2012).

The RT-PCR had the best performance in specificity, Positive likelihood ratio and positive predictive value in our present findings. However, it has lower sensitivity compared with NASBA-ELISA, indicating that the amplification efficiency of PCR is lower than NASBA as they all using the same RNA template. Lacking of standardization of PCR leaded to the failure to include DNA detection being incorporated into the EORTC/MSG criteria for the definition of invasive fungal infection.

The NASBA-ELISA was proved the most sensitive assay and had the best performance in negative predictive value, Youden index, and *К* value in our present findings. The high sensitivity of NASBA-ELISA is probably since that NASBA possesses a higher inherent amplification capability than RT-PCR because each cDNA template produces numerous RNA copies per cycle in the former technique while each cDNA merely doubles in number at each cycle in the latter. Further more, the detection procedure is conducted with enzyme-linked immunosorbent assay of DIG-labeled NASBA amplicons. NASBA is better to reveal IA infection status since the RNA template of NASBA amplification is closely related to active microbes. Since the NASBA assay only allows amplification of template nucleic acids with T7 promoter and thermal denaturation is absent for NASBA, contaminating background of homologous DNA is not a problem. As an alternative technology to detect NASBA amplification products, ELISAs avoid subjective interpretations of amplification results due to “bands of unknown origin” or “nonspecific products” among other features and can detect batch of samples automatically. Moreover, though NASBA-ELISA is a technique of end-point amplification products analysis, it also allows for quantification in a certain range of original RNA concentrations that cover all the significant amount of *Aspergillus* in clinical samples.

In this study, we developed a simple quantitative microplate hybridization method for detection of NASBA products and evaluated its performance by plasma sample of patients at high risk for IA. The results exhibited that NASBA-ELISA has several advantages such as high sensitivity, good specificity (absence of cross-reactivity with other unrelated bacteria or fungus) and can be easily performed. It can analyze a numbers of samples simultaneously without any special instrument and reasonably it may be particularly useful for routine purposes in less developed laboratories. Combining NASBA-ELISA with other techniques can improve the diagnostic accuracy and could be particularly useful in specific clinical situations.

## Additional file

10.1186/s13568-016-0266-0 Additional table.

## Abbreviations

NASBA-ELSA: enzyme-linked immunosorbent assay of nucleic acid sequence-based amplification
IA: invasive aspergillosis
GM: galactomannan
GM-EIA: galactomannan by enzyme immunosorbent assay
*E.coli*: *Escherichia coli*
*C.albicans*: *Candida albicans*
*S. aureus*: *Staphylococcus aureus*
*P. aeruginosa*: *Pseudomonas aeruginosa*
*C.neoformans*: *Cryptococcus neoformans*
